# Biosensing Cytokine IL-6: A Comparative Analysis of Natural and Synthetic Receptors

**DOI:** 10.3390/bios10090106

**Published:** 2020-08-24

**Authors:** Eleonora Alfinito, Matteo Beccaria, Mariangela Ciccarese

**Affiliations:** 1 Department for Innovation Engineering, University of Salento, Piazza Tancredi, I-73100 Lecce, Italy; 2 Department of Mathematics and Physics “Ennio de Giorgi”, University of Salento, Piazza Tancredi, I-73100 Lecce, Italy; matteo.beccaria@unisalento.it; 3 National Institute for Nuclear Physics, I.N.F.N, Lecce Unit, I-73100 Lecce, Italy; 4 Oncology Unit “Vito Fazzi” Hospital-Lecce, I-73100 Lecce, Italy; mari.ciccarese75@gmail.com

**Keywords:** IL-6 cytokine, biosensors, Proteotronics, aptamers, topological analysis

## Abstract

Cytokines are a family of proteins which play a major role in the regulation of the immune system and the development of several diseases, from rheumatoid arthritis to cancer and, more recently, COVID-19. Therefore, many efforts are currently being developed to improve therapy and diagnosis, as well as to produce inhibitory drugs and biosensors for a rapid, minimally invasive, and effective detection. In this regard, even more efficient cytokine receptors are under investigation. In this paper we analyze a set of IL-6 cytokine receptors, investigating their topological features by means of a theoretical approach. Our results suggest a topological indicator that may help in the identification of those receptors having the highest complementarity with the protein, a feature expected to ensure a stable binding. Furthermore, we propose and discuss the use of these receptors in an idealized experimental setup.

## 1. Introduction

In the recent COVID-19 pandemic, anti-inflammation therapies have been recommended to reduce medical complications. As a matter of fact, subgroups of patients with severe forms of COVID-19 have developed hyperinflammation syndrome [1]. In particular, high levels of cytokine IL-6 pushed the clinical trials of specific inhibitors of this protein and of Janus kinase (JAK) on COVID-19 patients in China [1,2]. The improvement of patient health and the modest side effects suggest the use of IL-6 antagonist therapy not only against COVID-19 but also in the treatment of other lethal viruses [2].

The role of cytokines in several diseases, from rheumatoid arthritis to cancer, is well known [3,4,5]: IL-6 is synthesized in the initial stage of inflammation and moves to the liver through the bloodstream, followed by the rapid induction of an extensive range of acute phase proteins such as C-reactive protein (CRP), serum amyloid A(SAA), fibrinogen, haptoglobin, and a1-antichymotrypsin [6]. In view of the range of biological activities of IL-6 and its pathological role in various diseases, IL-6 targeting has constituted a novel treatment strategy for various immune-mediated diseases. The development of Tocilizumab was a direct result of this hypothesis. Tocilizumab is an IgG1 class anti-IL-6 R monoclonal Ab that was generated by grafting the complementarity determining regions of a mouse antihuman IL-6R Ab on to human IgG1 [7]. It blocks IL-6-mediated signal transduction by inhibiting IL-6 binding to transmembrane and soluble IL-6R. Recently, Tocilizumab represents an area of investigation in the clinical care of severe COVID-19 pneumonia [8,9,10].

The relation of inflammation with various types of cancer has been suggested by several studies. Recently, it has been showed that anti-inflammatory agents attenuate tumor growth in breast cancer-bearing mice [11]. Malignant cells exhibit a high proliferation, which can be enhanced by inflammation. The inflammatory molecules in the tumor’s microenvironment are mainly secreted by tumor cells themselves and/or other stromal cells [12]. IL-6 plays a critical role in the expansion and differentiation of tumor cells [13,14] and can also modulate the tumor’s therapeutic resistance, such as multidrug resistance (MDR) [15], whose engagement triggers the activation of JAK and the downstream effectors STAT3, SHP-2/Ras, and PI3K/Akt [16]. It has been revealed that IL-6 levels are significantly elevated in lung and breast cancer patients associated with poor prognosis; moreover, IL-6 can affect all aspects of the tumorigenesis process by regulating proliferation, apoptosis, metabolism, survival, angiogenesis, and metastasis [17]. Blocking IL-6 and/or its receptor could be a trigger point to cure tumors associated with high levels of this cytokine, as in breast cancer.

Several drugs have been produced to contrast IL-6 activity [18]. The detection of very small alterations of cytokine levels [19], and specifically of IL-6, could be helpful in the early recognition of an inflammatory state [20,21,22,23,24]. Biosensors may use aptamers, antibodies, and also antibody fragments (Fabs) as detection elements [25]. Their performances, pros, and cons have been recently discussed in [25]: Aptamers usually show a very high affinity to the ligand and have a very small size, which allows them to produce a high density sensing element, thus reducing the LOD (limit of detection) of the detector. On the other hand, Fabs have the benefit of cost effective processes and fast production, although, in general, they show lower affinity than aptamers [25].

While the concept of affinity is qualitatively simple to understand, it is not uniquely defined at the quantitative level. Often, it is given in terms of the dissociation constant K_D_, defined as the ratio of the two dynamic constants k_off_/k_on_, the off/on rate constants, which are themselves used to measure affinity. Specifically, K_D_ describes the dynamic equilibrium of two species which combine in a product (the complex) and of the product which decays in the two species. Generally speaking, the smaller the K_D_ value, the higher the affinity. On the other side, the role of k_off_ [26,27], which signals the life-time of the complex, is becoming even more relevant, in particular in pharmaceutics, due to the performance discrepancy between drugs tested in vitro/silico or in vivo [26].

Finally, we are interested in understanding whether the best-fitting arrangement of the receptor-ligand pair can be quantified. For this purpose, IL-6 offers an uncommonly wide set of different choices (receptors), between aptamers [20,21,28] and Fabs [22,23]. Aptamers are relatively young synthetic biomolecules [29] made of single strands of DNA or RNA selected in vitro to bind with high affinity a specific ligand (proteins, viruses, ions). Besides, they have outstanding performances in terms of affinity and stability, both in vitro and in vivo [30,31]. Furthermore, the interest in pharmaceutics [32] has pushed forward the investigation of their 3D structure with crystallographic analysis [28,33,34] and computational analysis [35,36,37].

Fabs are antibody fragments, produced in labs with cloning techniques with good ligand affinity. In general, they are less customizable than aptamers and are more prone to denaturation, although, at present, they remain the best choice for those who need cheap and quickly available material [25].

To shed light on the mechanisms of binding, the 3D structures of some of the IL-6 receptor complexes have been resolved using crystallographic analysis [34]. Remarkable differences in the way receptors bind the ligand have been highlighted. The aim of the present investigation is to compare these receptors on a totally topological basis in order to identify their features, performances, and limits in the perspective of their utilization in sensing devices. The proposed method takes advantage of public and freely available data [34] and of a theoretical approach known as Proteotronics [38]. At present, databases include information for IL-6 in its native state, or in a complex with different receptors. The list of the structures is shown in Table 1, and, besides the natural IL-6∙IL-6R∙gp-130 assembly, it contains the cytokine complexed with some Fabs and a Slow-Off-Rate (low dissociation rate with the ligand) Modified aptamer (SOMAmer) [28]; this novel kind of aptamer has been designed to implement the affinity to ligands, specifically to those usually quite difficult to bind [39].

The choice of the most appropriate receptor for a biosensor is often biased by the experience that researchers have with it. This may happen for economic reasons and also because the expected performances of different receptors are difficult to compare. Here we outline a procedure to attempt such a comparison, at least within a specific theoretical approach, by analyzing the topological features of different receptors complexed with IL-6.

To this aim, we use a theoretical approach [38] which translates the main topological elements of the assigned biomolecule (a protein, an oligonucleotide, a complex) into a complex network taking into account the degree of interaction between the parts of the biomolecule itself.

By complex network we mean a graph made of nodes and links: one node for one nucleotide/amino acid, and one link for a pair of nearest neighbor nodes [40].

After this mapping, the resulting complex network can be analyzed in terms of its transport properties. To this aim, each link is associated with specific impedance elements [38] and an electrical current is allowed to flow through the network. This kind of theoretical probing is able to detect the internal structure of the network and, in particular, to reveal the presence of short-paths or bottlenecks [37,38]. In the present case, the spreading of the current flow aims to detect the quality of the binding. We have compared the receptors listed in Table 1 and introduced an indicator that is able to sort the topological complementarity with the ligand (IL-6). As expected, the clinical Fab is one of the best performing options, although another Fab as well as the SOMAmer can compete with it. Finally, we propose predictions about the expected performances of these receptors when used in an ideal sensor.

## 2. Materials and Methods

### 2.1. Materials

All data concerning the present investigation is available in the public data bank repository, hereafter called PDB [34]. In the following we give a sketch of each of them:

IL-6

IL-6 cytokine belongs to the family of gp130-coupled cytokines, i.e., proteins that transfer the signal through this glycoprotein, also known as the β-receptor. Activation of IL-6 forms a dimer with the α-receptor IL-6R, at Site I, then a hetero-trimer, binding gp130 at Site II [41]. Finally, the quaternary structure is obtained when two hetero-trimers become attached (Sites II and III) [41]. It has been postulated that only in the hexameric quaternary structure is IL-6 able to function properly [41,42]. Several artificial receptors have been tested against IL-6, IL-6R, and gp-130 [18], and some of those entered and passed clinical trials [7,8,9,10,18,28,43,44]. All of these receptors show high affinity to the ligand and have specific inhibitory abilities. Both affinity and inhibition are the results of many factors, and an important one is the level of topological complementarity. In the typical scenario, IL-6 and its receptors undergo a conformational change when they bind together, and the cytokine seems to be able to assume different forms, adapting its shape to the receptor [18]. For example, a massive conformational change has been claimed at the origin of the inhibition mechanism of IL-6 due to the clinical antibody Olokizumab (OKZ). Indeed, the binding process forces some coil-shaped amino acids of the cytokine to deform into a small helix that occludes the gp130 binding site (Site III) and inhibits the hexamer formation [18]. In this case, inhibition seems to have a peculiarly steric origin.

The 3D structure of this protein has been resolved in the native state [45], i.e., the protein alone in the minimum of conformational energy, and also complexed with several receptors [18,26,28,29,32]. It is made of 4 main helices, sequentially named A, B, C, and D, and about 20 amino acids long, and a 5th small helix, say E, of 12 residues. In the present analysis, we refer to the *active* state as the state the protein assumes when complexed with a receptor [46]. In general, active and native state refer to different protein conformations, and, specifically in IL-6, the shape of the active state depends on the receptor.

IL-6R

The α-receptor of IL-6 (IL-6R) is a 3 domain protein (D1, D2, D3) and has been expressed and resolved completely in [47], in the absence of any ligand (native state), and also complexed with IL-6R and gp130 [41]. IL-6R is itself relevant for disease detection since increased levels can be found in patients suffering from different inflammatory illnesses [48]. Domain D1 is characteristic of the Ig superfamily [47], and thus relevant for the design of anti-IL-6R antibodies [7]. On the other side, crystallographic data concerning IL-6R bound to IL-6, specifically in the heterotrimer [41], contain only two of three IL-6R domains, i.e., D2 and D3. The missing information about D1 is probably due to its position (in the extracellular region). We cannot discuss cases where the native vs. active differences are due to changes in the D1 domain.

SOMAmer SL1025

SOMAmer (Slow Off-rate Modified Aptamer) is a new kind of DNA aptamer obtained by introducing modified moieties in the standard nucleotide catalog. This characteristic is of high interest in producing effective drugs, because it avoids metabolic degradation and a rapid clearance [48]. SOMAmers are effective against many ligands and also proteins usually considered challenging [39,49]. In particular, SOMAmer SL1025 [43,50] binds with high affinity (K_D_ = 0.2 nM) to human IL-6 protein and blocks the binding with IL-6R [51]. It is efficacious against rheumatoid arthritis in animal trials [50]. The 3D structure of SL1025 complexed with IL-6 has been deposited in two different entries, named 4ni7 and 4ni9 [28]. They are very similar and the latter is the most complete. Therefore, we limit our study to 4ni9 (chain A for the cytokine and B for the aptamer). The aptamer induces a poor or null conformational change in the protein, although it is presumable that it strongly adapts its native shape to obtain a large complementarity with the protein.

Olokizumab (OKZ)-Fab Portion

Olokizumab is a humanized monoclonal antibody, whose Fab fragment has been resolved and is available in PDB 4cni [18]. It has a very high affinity to the cytokine IL-6 (K_D_ = 10 pM). This high affinity is due to the high level of interconnection between the two biomolecules. The presence of OKZ induces a conformational change in IL-6. Crystallographic analysis revealed a noticeable difference with respect to the natural assembly of IL-6R∙IL-6∙gp130. Here, a set of few amino acids from GLU42-ASN47, detected in other complexes as a random coil, appears organized in a small helix (helix F). This small helix occludes the gp130 receptor-binding pocket, thus producing the high inhibitory activity. At present, OKZ is in a Phase III clinical trial with patients with rheumatoid arthritis [52].

**Table 1:** List of public data bank repository (PDB) [34] entries used in the present paper. Entries 1alu and 1n26 refer to the native states of IL-6 and IL-6R, respectively.

#Entry	Ligand	Receptor	K_D_(pM)
1p9m	IL-6	gp130, IL-6R	80(IL-6R∙gp130) -900(IL-6R) [41] *
1alu	IL-6	n.a.	--
1n26	n.a.	IL-6R	--
4zs7	IL-6	Fab 68F2	13–21 [44]
4cni	IL-6	Fab OKZ	10 [18]
4o9h	IL-6	Fab 61H7	3.3–6.3 [44]
4ni9	IL-6	SOMAmer SL1025	200 [51]

* data refer to the trimeric structure.

*61H7 & 68F2- humanized Camelid-Fab*

**Figure A1:**
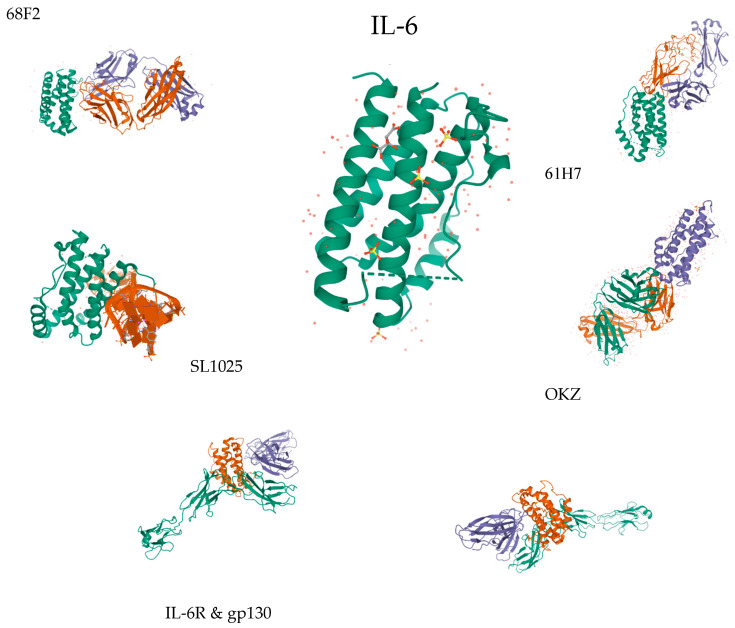
The 3D pictures of IL-6 alone (in the middle) and conjugated with different receptors. The complex IL-6∙ gp130∙ IL-6R is given in two different orientations (IL-6R in blue and green, gp130 in green). Source: The Protein Data Bank [34].

61H7 and 68F2 are neutralizing IL-6 antibody fragments. Antibodies were obtained upon Llama immunization with human IL-6 and exhibit ultra-high affinity to the cytokine, with a K_D_ = 3.3–6.3 pM (61H7) and K_D_ = 13–21 pM (68F2) [44]. On the other hand, the way 61H7 and 68F2 bind IL-6 is quite different: both were selected to block Site I of the cytokine, i.e., mimicking the action of IL-6R, but while 61H7 binds the top of the IL-6 helix bundle, 68F2 is set transversally toward the same side (see the Appendix A, Figure A1).

### 2.2. Methods

We used an investigation technique named Proteotronics [32,36,37,38,53] which explores the electronic responses of biomolecules by using a complex network approach. The technique is intuitive and simple to use. The main idea is to map the biomolecule’s tertiary structure (3D) into a graph, which accounts for both its structural and physico-chemical properties. Each node corresponds to a nucleotide/amino acid and contains all the information we need to account for the interaction we have to describe (barycenter position, resistivity, temperature, dielectric constant, elastic module, and so on). Undirected links may connect two nodes. This happens depending on the entries of the distance matrix describing the graph [38]. It is a zero-diagonal symmetric matrix whose dimension is given by the number of nodes and (*i,j*)-th entry equal to the distance between the *i*-th and *j*-th nodes. In this modeling, the presence of an activated link represents the existence of an interaction between the nodes. Because each known interaction is distance-sensitive, a link is drawn only if the nodes are closer than the assigned cut-off distance, *D*. In such a way, we can figure the nodes like soft spheres of radius *D*/2 that interact only when they overlap. The connected nodes are the nearest neighbor nodes, the only ones able to interact. When the parameter *D* is increased, the number of links also increases. The distance matrix is therefore mapped into an adjacency matrix, *A*, which scores zero when the distance between the *i,j* nodes, *l_ij_*, is larger than *D*, otherwise scoring 1. The graphical representation of *A* is called a contact map (CoMa), gives a prompt sketch of nearest neighbor nodes, and describes the skeleton of the biomolecule.

**Figure 1:**
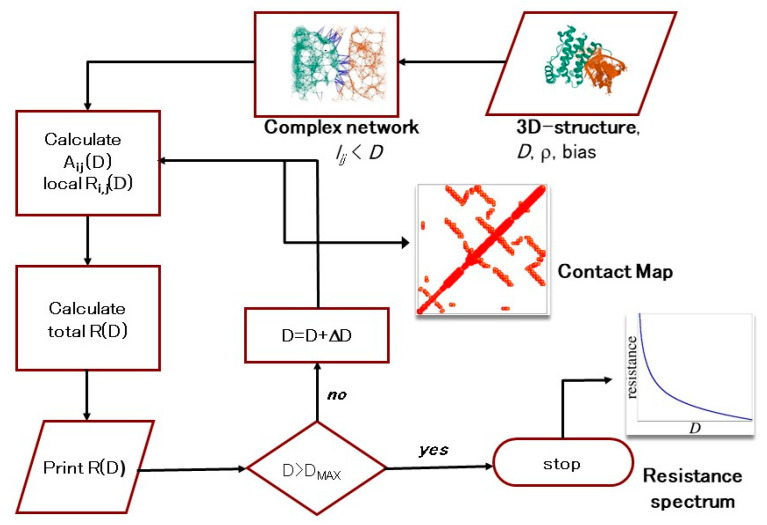
Flow chart of the computational procedure giving the resistance spectra and the contact maps. Starting from the 3D structure of the biomolecule and assigning a set of input parameters (D, *ρ*, bias), the equivalent complex network is built up. The adjacency matrix A is generated, and the contact map ensues. Local resistances are calculated, according to Equation (1), and from them we obtain the resistance of the whole network. The process is iterated over a large range of D values. Finally, the resistance spectrum is produced.

In the present analysis, we are interested in electronic interactions, and therefore an elementary impedance was associated to each pair of connected nodes, as follows [38,53]:

(1) $$z_{ij}\left(\omega \right)=\frac{l_{i,j}\times \rho_{i,j}}{S_{i,j}\left(1+i\rho_{i,j}\varepsilon_{0}\varepsilon_{i,j}\omega \right)},\;\;\;\;\;\;\;\;\;\;\;\;\;\;\;\;\;\;\;\;\;\;\;\;\;S_{ij}\left(D\right)=\frac{\pi \left(D^{2}-l_{i,j}{}^{2}\right)}{4}$$

where *ρ_i,j_*, ε*_i,j_*, and *S_i,j_* are the resistivity, the relative dielectric constant, and the intersection area of the pair of connected nodes, respectively. The resistivity and relative dielectric constant may be calculated as described in [53]. Notice that the electrical response, as defined in Equation (1), depends on the biomolecule 3D structure. Impedance reduces to the simple resistance, *r_ij_*, when ω = 0. The network total impedance, *z(ω)*, is calculated by solving a set of linear equations resulting from the application of the Kirchhoff’s node rule (see, e.g., [38]). Finally, by allowing the value of *D* to vary, the impedance/resistance spectrum is produced (Figure 1). Often, in the present investigation, we use the *relative* resistance spectrum *rr(D)*, i.e., the ratio of two resistances (see below) calculated for increasing values of *D*. A schematic representation of the procedure is reported in Figure 1. 

## 3. Results

In this section we first describe the topological structures in terms of contact maps. We then introduce a new (topological) indicator that may be used to characterize the quality of binding and that we call ToCI. Finally, we propose an ideal experimental setup (i.e., a *Gedanken-Experiment*).

### 3.1. Contact Maps

**Figure 2:**
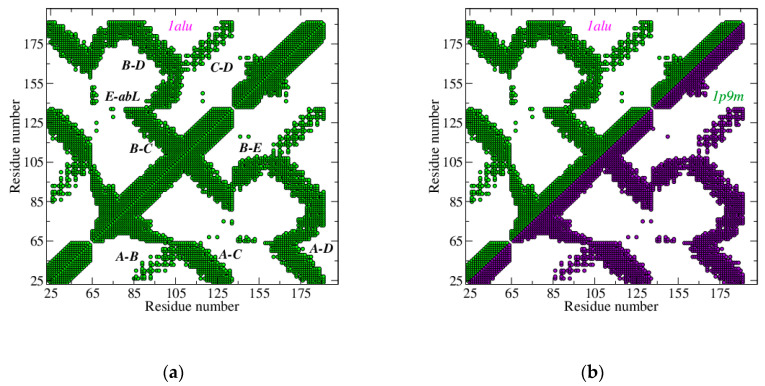
Contact maps of IL-6, in: (**a**) the native state (PDB ID: 1alu [45]) and (**b**) the active state due to the conjugation with natural receptors IL-6R and gp130 (PDB ID:1p9m [41]), *D* = 15 Å. (**a**) The intra-contacts among helices A, B, C, D, and E are highlighted, and *abL* indicates the loop between helices A and B; (**b**) IL-6 in the native state (green) is reported vs. IL-6 in the active state (violet).

As we already mentioned, in our approach the interaction network is represented by its contact map (CoMa). A CoMa is a picture of the nearest neighbor nodes for an assigned value of *D*. Each point of coordinates (*i,j*) is drawn only if the corresponding nodes *i* and *j* are connected. The number of connected nodes grows with *D*, reaching the maximum value, M(M-1)/2 (M is the number of nodes), for high values of *D* [38]. Furthermore, the plot of connected nodes is specific for each biomolecule and helps to identify its secondary structure (Figure 2a). Protein IL-6 adapts its shape to the receptor, as is revealed comparing the CoMas of its native and active state. Each CoMa corresponds to the network adjacency matrix, which is symmetric. Therefore, it can be drawn completely (full picture), or only considering *i < j* (or *j < i*) (half picture): in the latter case, the other half of the picture is used to draw another adjacency matrix.

**Figure 3:**
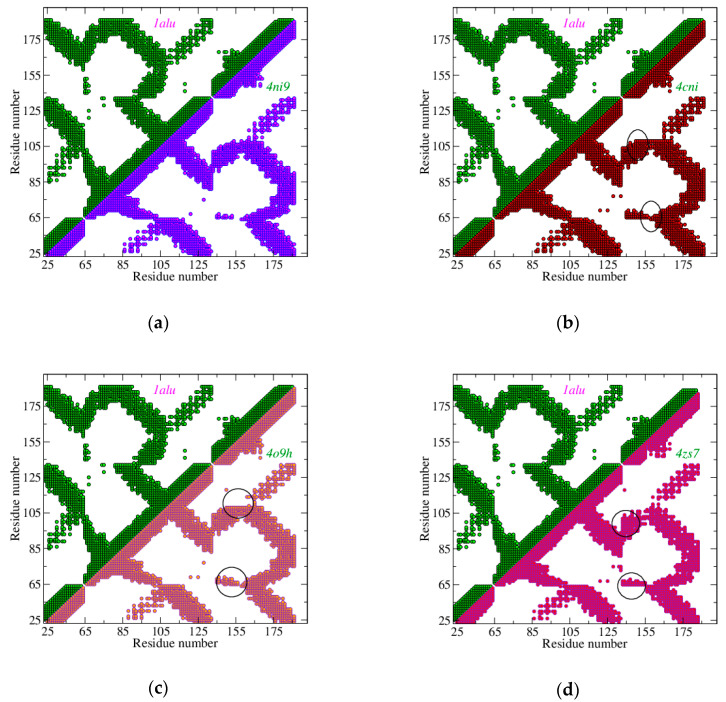
Contact maps of IL-6, in the native state and the active state, due to the conjugation with different receptors, *D* = 15 Å. (**a**) IL-6 in the native state (green) vs. IL-6 in the active state due to the conjugation with SL1025 SOMAmer (blue); (**b**) IL-6 in the native state (green) vs. IL-6 in the active state due to conjugation with OKZ Fab (red); (**c**) IL-6 in the native state (green) vs. IL-6 in the active state due to conjugation with 61H7 Fab (pink); (**d**) IL-6 in the native state (green) vs. IL-6 in the active state due to conjugation with 68F2 Fab (magenta). Main differences with the native states have been marked with an ellipse.

For example, in Figure 2a we report the IL-6 CoMa (full picture) in its native state (PDB ID: 1alu [45]), while in Figure 2b the half map of the native state is compared with the half map of IL-6 in the active state produced by the conjugation with its natural receptors, IL-6R and gp130 (PDB ID: 1p9m [41]). This choice has also been adopted for Figure 3, where the active states of IL-6, due to the conjugation of SOMAmer SL1025 (PDB ID: 4ni9 [28]), OKZ (PDB ID: 4cni [18]), and two humanized Camelid Fabs (PDB ID: 4o9h [43]), and (PDB ID: 4zs7 [44]) have been compared with the IL-6 native state. This comparison suggests modest differences between the native and active state of IL-6 bound to its natural receptors or with SOMAmer.

On the other side, Fabs are found to induce a more relevant conformational change in the protein, mainly due to helix E. This helix shifts toward the AB loop and away from helix C. For a faithful comparison, the same strands of IL-6 have been used for all the entries, both in Figure 2 and Figure 3—in particular, a set of 133 of amino acids from GLU 10 to MET 171. Of course, differences due to protein moieties not present in this set cannot be detected.

**Figure 4:**
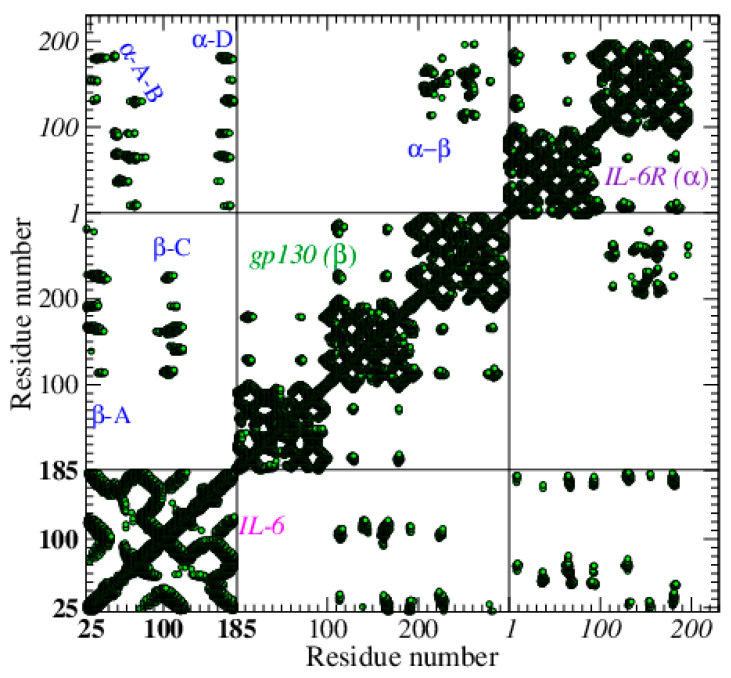
Contact map of IL-6 complexed with its natural receptors, IL-6R, the α-receptor, and gp130, the β-receptor, PDB ID: 1p9m [41], *D* = 15 Å. The three boxes on the diagonal contain the intra-contacts of IL-6, gp130, and IL-6R, respectively. The outside contacts (between protein and receptors) are in the lateral boxes. Some of the best resolved receptor (α/β) –protein contacts are highlighted. Progressive node numbers are indicated for IL-6 (in bold), gp130, and IL-6R (in italic).

**Figure 5:**
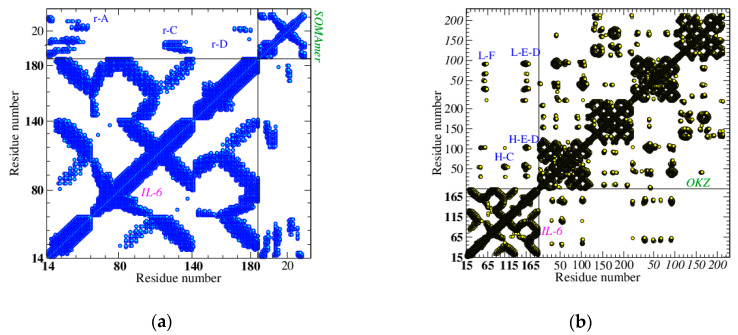

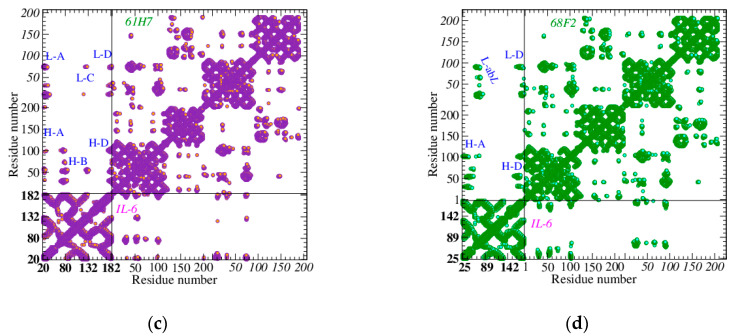
Contact maps of IL-6 complexed with different receptors, *D* = 15 Å. (**a**) SOMAmer SL1025: main links between the receptor (r) and helices A, C, and D are indicated; (**b**) Fab OKZ: main links between four IL-6 helices, C, D, E, F and the heavy (H) and light (L) chain of the receptor are indicated; (**c**) Fab 68F2: main links between IL-6 helices A and D and the heavy (H) and light (L) chain of the receptor are indicated; (**d**) Fab 61H7: main links between IL-6 helices A and D and the heavy (H) and light (L) chain of the receptor are indicated. Helices B and C show contacts with chains H and L, respectively. Progressive node numbers are indicated for IL-6 (bold), receptor heavy chain (normal), and receptor light chain (italic).

When the complexes are analyzed, the differences among them appear much more relevant. Using the natural trimer as a benchmark, its CoMa, taken at *D* = 15 Å, shows a high number of interlinks with both receptors. In particular, IL-6R (α-receptor) has a preferential binding with helices A, B, and D, while gp130 (β-receptor) appears more close to helices A and C (see Figure 4). The SOMAmer elicits a very similar kind of binding and, despite its small size, spans a very large number of links with IL-6 (mainly with helices A, C, and D) in a very good mimicking of the natural receptors (see Figure 5a).

Both chains H and L contribute to the binding, in all Fabs. All Fabs bind helix D, and the two Camelid Fabs also bind helix A; small helices E and F have a main role in the complex with OKZ. Fab 61H7 has a huge number of links with helices B and C.

### 3.2. ToCI

The topology of the biomolecule (receptor, ligand, or their complex) is investigated through its equivalent network [38]. A pair of ideal contacts is used to connect the network to an external battery, working in D.C., and in the linear regime [37,38,53]. In this way, an electron current flows inside the biomolecule equivalent network, thus detecting its internal structure [41,42]. In doing so, the relative resistance spectrum, *rr* ≡ *rr* (*D*), where rr is the ratio of the calculated resistance of the IL-6∙receptor complex to the resistance of IL-6, has been introduced as an investigation tool.

**Figure 6:**
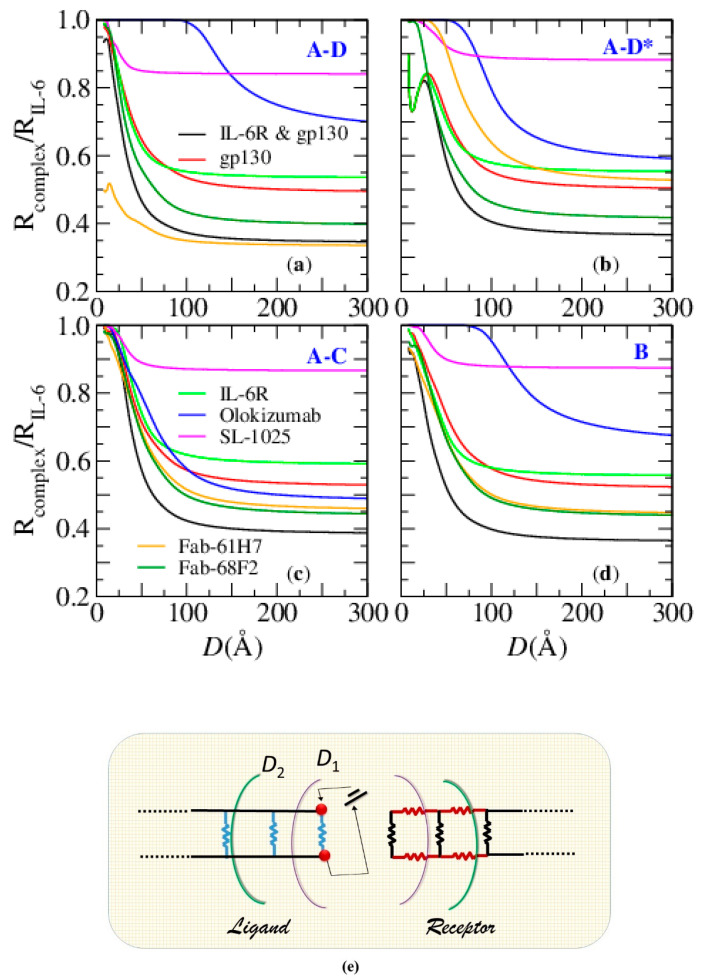
Relative resistance of IL-6 complexed with several receptors. Ideal electrical contacts are: (**a**) on helices A–D close to N-terminus, input/output GLU10/MET171; (**b**) on helices A–D in the opposite orientation (A–D*), input/output THR30/ASN142; (**c**) on helices A–C, input/output THR30/ALA117; (**d**) on helix B, input/output ASN66/SER95; (**e**) the ladder resistance network equivalent of the complex.

Focusing on the topological information probed by the flow, we do not account for the specific electronic properties of each node. Instead, we assume the same value of resistivity for all of them (here *ρ* = 10^14^ ΩÅ). Specifically, by putting the electrical contacts on IL-6 (hereafter, the ligand), it becomes the source and the sink of an electrical current that flows through the receptor, the ligand, and the links between receptor and ligand (outside links). The dependence of the ligand resistance on *D* is simply accounted for when an electrical ladder representation is introduced (see Figure 6e): The ligand can be described as a ladder of resistances (L1) connected on one end to the external battery; increasing D, new branches are added to the opposite end, and the total resistance lowers [40]. Adding the receptor is equivalent to inserting another resistance ladder (L2) in parallel with the first one, and, therefore, the resistance of the complex is always smaller than that of the ligand. L2 accounts for intralinks (links inside the receptor) and outside links. As a matter of fact, it depends on the receptor size: by increasing D, the number of intralinks grows, and the L2 resistance decreases. At the same time, the number of outside links also grows, further lowering the resistance of L2. Finally, a large receptor may show a smaller resistance than a small receptor, i.e., its asymptotic (D large) rr value can be much smaller than 1. On the other side, outside links strongly affect *rr*, because they further reduce the resistance. This is the case of Fab 61H7, that, although smaller than IL-6R∙gp130, reaches a lower rr asymptotic value (see Figure 6a). Finally, SOMAmer SL-1025 is smaller than 1/10 of Fab 61H7. Accordingly, it has a higher *rr* value, although not as high as the size effect might suggest: in this case, multiple outside links contribute to reducing *rr*. In conclusion, the asymptotic value of the relative resistance spectrum is due to both the receptor size and the outside link.

Outside links are more numerous in complexes with high complementarity. The rapidity of outside links formation gives the rapidity of the *rr* decreasing, and therefore a fast rr decrease signals high complementarity.

The *rr* spectrum also contains other useful information about the binding: at the smallest *D* values, the ratio rr shows its maximum, that can be as large as 1, thus signaling a very small number of outside links, or a small growth toward a maximum smaller than 1 (see, for example Fab 61H7, Figure 6a). In the latter case, the rate of the growth of outside links is similar to that of intralinks (inside IL-6) [36], thus suggesting a complex made of two very close parts.

The calculation of rr has been performed by using four different contact positions, to account for the different ways the receptors bind the protein. In particular, Site I is the epitope to which IL-6R binds IL-6 and is roughly rendered by putting the contacts on helices A and D (GLU10-MET171) on the side of the N terminal. Site II, where gp130 binds IL-6, is rendered putting the contacts on the same A and D helices, but on the opposite side (THR30-ASN142). Due to the primary role of helix C in the natural binding of IL-6, a contact pair is also put on helices A and C (THR30-ALA117), and, finally, as an intermediate position, both contacts are put on helix B (ASN66-SER95). The profile of *rr* as *D* is varied and depends on the contact positions, showing in some cases very dramatic differences: in those cases, the corresponding complexes are much more asymmetric than the others. This is the case of Camelid Fab 61H7 [43,44] and of OKZ [18]: 61H7 Fab binds IL-6 at Site I, and therefore the contacts on the first position (Figure 6a) give a very low value of the maximum of rr, a sharp decrease, and a low asymptotic value (larger flux inside the receptor than in the ligand). Conversely, when contacts are put in the second position (Figure 6b), a larger value of the maximum, a smoother decreasing, and a higher asymptotic value (i.e., a smaller current in the receptor) are found. A specular result is obtained for OKZ, which targets Site II. Finally, neither contacts on helix B nor C improve the responses given by contacts put on helices A and D. Similar arguments explain the behavior of the other ligand∙receptor complexes (see Figure 6).

To quantify these differences, also collecting information about the maximum (closeness of parts), shape (complementarity of parts), and asymptotic value (role of size and outside links), we have introduced a quantity that we named ToCI (topological complementarity index). We calculated the area of rr under the sensitive height and over the sensitive range of *D*, i.e., the equivalent surface. The sensitive height is the difference between the value of *rr* for each *D* and its asymptotic value, and the sensitive range of *D* is the range from the minimum value of *D* (here 8 Å) to the value corresponding to 90% of the maximal sensitive height. Finally, to account for the different sizes of the complexes, the area was divided by the ratio of the complex/protein number of nodes. With such a definition, we aimed to take into account both the maximum value of rr and the rapidity of its decrease as well.

The value of ToCI is smaller when the contact position mimics that of the binding site. Specifically, three of the four non-primeval receptors had very similar performances, namely Fab 61H7 (1.2) and SOMAmer SL1025 (1.6), when bound to Site I, and OKZ (1.9) when bound to Site III (see Table 2). In some cases, as 61H7 and OKZ, the differences between data obtained putting the contacts on the correct position or the opposite, are very large. Finally, these results agree with the known very high affinity to IL-6 (see Section 2.1). The very good topological complementarity between these three receptors and IL-6 suggests that both SOMAmer SL1025 and Fab 61H7 could perform (at least when used to detect IL-6) as well as OKZ.

### 3.3. Gedanken-Experiment

A real biosensor is a customized and complex device whose performances mainly depend on the sensing element, signal transduction process, and amplification. Therefore, the description of an ideal sensor may appear unfeasible. Nevertheless, some common features may still be identified and make such a description meaningful. As a general requirement, a low limit of detection (LOD) is desirable. This is expected to imply a high affinity to the analyte. High affinity is a necessary condition to have a good selectivity. All the analyzed receptors have high/very high affinity to IL-6 and are thus potentially good candidates. Besides a low LOD value, we also desire a good sensitivity, i.e., a wide dose-response range. This means that the device (and in particular the receptors) gives sensitively different responses in a wide set of analyte concentrations. The way the sensor translates the capture action is specific to the sensor itself. Here, we focus on electrical (impedance or resistance) responses. In electrochemical sensors, a relevant measurement is that of the impedance spectrum. In particular, the zero frequency impedance value is associated to the so-called charge transfer resistance, R_CT_. Different doses are associated with different values of R_CT_. In quite different devices, for example FET/AFM devices [54], the analyte may be detected by conductance measurements instead of resistance ones.

Here, we compare the resistance of a biosensing element made of the previously investigated receptors (61H7 and 68F2 Fabs, OKZ, IL-6R, and SL1025) and try to predict the response when the concentration of the analyte increases (the dose response). Of course, speculations strictly apply to an ideal biosensor, i.e., such that the receptors are perfectly oriented, the noise/signal ratio is negligible, the electrode is perfectly functionalized, the response is completely reproducible. Nevertheless, our arguments are expected to establish bounds on the receptor performances.

Let us now describe the behavior of the complex as the parameter *D* is varied. In our modeling, it will be ideally traded for the analyte concentration. The resistance of the biosensing element, R_sample_, is given in terms of the resistances of the single receptor-ligand complex, r_comp_, and the resistance of the receptor, r_rec_ [53,55,56]. Specifically, when the receptors of the sample receive the analyte, a fraction f binds the ligand. The extreme case *f* = 0 means that the sample resistance is totally due to the receptors, while *f* = 1 means that the sample resistance is totally due to the complexes. Furthermore, the storm of analytes strongly affects both receptors and complexes, raising their conformational energy [46]. In other terms, bonds become weaker and, in general, biomolecules are less rigid [46]. In our model, a bond reduction means a reduction of the value of *D*. Therefore, the complete variation of f covers the range (*D_0_, D_1_*), *D_0_* > *D_1_*, with *D_0_* representing the condition *f* = 0 and D_1_ representing the condition *f* = 1. In other terms, each dose corresponds to a specific *D* value [53,55,56]. The total sample resistance reads

(2) $$R_{sample}\left(D\right)=N\left[f\times \left(r_{comp}\left(D\right)\right)+\left(1-f\right)\times (r_{rec}\left(D\right))\right]$$

where *N* is a numerosity factor, e.g., it is the number of receptors in an ideal linear arrangement; more generally, it is the effective number of receptors as seen by the electrical contacts. The fraction of complexed receptors also depends on *D* [53,55,56].

The ideal LOD is given by a single capture process, *f = 1/N*, i.e., the activation of the smallest part of the biosensing element:

(3) $$R_{sample}\left(D_{LOD}\right)=\left(r_{comp}\left(D_{LOD}\right)\right)+\left(N-1\right)\times (r_{rec}\left(D_{LOD}\right))\;\approx \;\;\;\;\;N\times r_{rec}\left(D_{LOD}\right)$$

where r_comp_ (D_LOD_) is assumed to be smaller than *N*x r_rec_(D_LOD_).

Finally, when the dose is high, all the receptors bind the ligand, and (*f* = 1):

(4) $$R_{sample}\left(D_{1}\right)=N\times r_{comp}\left(D_{1}\right)$$

Measurements of electrochemical impedance [20,21,53] often detect a growth of *R_sample_*, due to the injection of the analyte. From Equations (3) and (4), this means, for example, that *r_rec_*(*D_LOD_*) is smaller than *r_comp_*(*D_1_*). On the other hand, assuming *r_rec_*(*D_LOD_*) > *r_comp_* (*D_LOD_*), it also means that:

*r_comp_ (D_LOD_) < r_rec_(D_LOD_) < r_comp_(D_1_)* (5)

**Figure A2:**
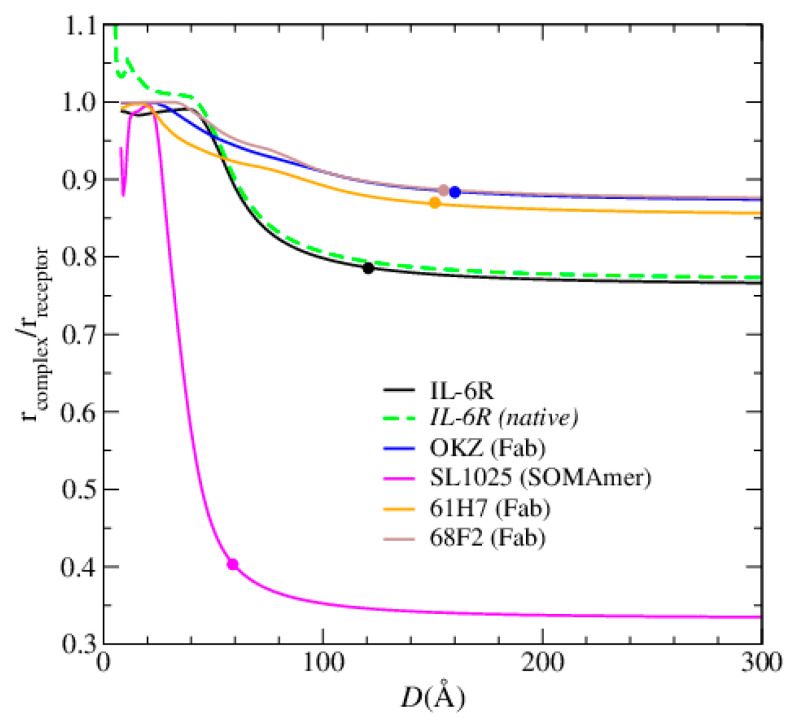
Relative resistance of IL-6 complexed with different receptors; contacts on the receptor. The dashed line describes the relative resistance of IL-6 complexed with IL-6R vs. IL-6R in its native state (PDB ID: 1n26 [47]).

When *D_LOD_* > *D_1_* (see Equation (1)), i.e., increasing the dose value, *D* shifts toward lower values, in agreement with previous speculations. Finally, the wide range of *R_sample_* variation (the sensibility) is given in terms of the single receptor/complex resistance (Equations (3) and (4)). The exact range of *D* can be matched to the experiments. For the purposes of this section, i.e., with the aim of comparing the sensibility of different receptors, we can arbitrarily choose it as the widest possible. Specifically, here we select the *sensitive D* range, introduced in Section 3.1, and perform the calculation of the complex resistance by putting the contacts on the receptor in order to mimic the real case (see Figure A2). This range surely overcomes the range in which any possible real device can work and has to be considered a guideline to estimate the real behavior of the considered receptors.

A final remark about Equation (5): the constrain *r_rec_(D_LOD_) > r_comp_ (D_LOD_)* is straightforward when *r_rec_* is the resistance of the receptor in its active state (appropriately deformed to accommodate the protein). On the other hand, *r_rec_* should be meant as the resistance of the receptor in its native state (ligand-free). Among the selected receptors, the native state is known only for IL-6R, and, in general, the 3D structure of a biomolecule is known both in its native and active state only in few cases. On the other hand, for large *D* values, the resistances of the native and active states are very close simply because both networks have the same node number. In this regime, we will be allowed to equate r_rec_ with the resistance of the receptor in its active state, and this justifies Equation (5).

Finally, the device sensibility is estimated by the difference (Δ) and the ratio (κ) of the microscopic resistances:

(6) $$k=\frac{R_{sample}\;\left(D_{1}\right)}{R_{sample}\left(D_{LOD}\right)}=\frac{r_{comp}\left(D_{1}\right)}{r_{rec}\left(D_{LOD}\right)}$$

and:

(7) ΔN=1N(Rsample(D1)−Rsample(DLOD))=rcomp(D1)−rrec(DLOD)

We found that SOMAmer SL1025 and Fab 61H7 have very high resistances, and also a very high resistance increment, Δ, (see Table 3). On the other side, SL1025 has the smallest relative ratio, κ, and Fab 61H7 the highest. In conclusion, both are able to convert the ligand capture in a large electrical response. In particular, Fab 61H7 can be used to explore a wider range of analyte concentrations, while SL1025 is able to give a strong signal also at the earliest capture event. 

## 4. Discussion

Several diseases are accompanied by inflammatory states and high levels of cytokines. It is the case, for example, of cancer, where both malignant cells proliferation and therapeutic resistance are enhanced by inflammation. In breast cancer, for example, cancer-associated adipocytes trigger radio-resistance by secreting IL-6 [57]. Therefore, targeting the IL-6/JAK/STAT3 pathway can be considered as an effective therapeutic approach for cancers associated with overexpression of IL-6, including breast cancer [13,58,59,60].

Most recently, COVID-19 patients in severe conditions also benefitted from these therapies [8,9,10]. Therefore, the development of devices for the detection of altered levels of IL-6 in low symptomatic people is desirable for prevention and early detection of the insurgency of inflammation condition.

We have analyzed some IL-6 receptors, with the aim of revealing their possible performances when used as the active part of an IL-6 biosensor. We have compared some topological characteristics of the associate interaction networks. A very interesting representation of these networks is given in terms of their contact maps, CoMas, which represent the closest nodes, for an assigned cut-off distance. By analyzing the CoMas, it is possible to observe that the smallest receptor, a SOMAmer, made of 32 nodes, is able to set up a network of bindings as wide as those of the other receptors, which instead are made of about 400 nodes. Furthermore, the SOMAmer pattern of links is very similar to that produced by IL-6 with its primeval receptors, IL-6R and gp130. Specifically, it shows many links with helix C, bound to gp130 in the natural assembly, which are poorly present in the other receptors. The CoMas of the analyzed three Fabs (two from Camelid antibodies, 61H7 and 68F2, and one from a therapeutic antibody, Olokizumab-OKZ) reveal some specific features: 61H7 mainly binds the longest IL-6 helices (A, B, C, D); OKZ has several links with the short helices E, F; finally, 68F2 binds helices A, D, and the loop between A and B. These differences are due to the different orientations of the receptors toward IL-6, which, on the other side, reflect the different inhibition mechanisms activated by the receptors.

This analysis has been complemented with a percolation-like procedure which allowed us to analyze the surface complementarity of the receptor-ligand pair. With this aim, we have introduced an indicator named *ToCI* (topological complementarity index) and ranked the receptors accordingly. Most of the analyzed receptors performed better than the natural assembly, and this result agrees with their high affinity to the cytokine. Finally, we have compared the expected resistance response of IL-6 when complexed with the analyzed receptors and have endorsed the use of Fab 61H7 and SOMAmer SL1025 for an electrochemical biosensor. The strategy that we have described is straightforward to implement and may be used to compare different receptors of a specific ligand, when their 3D structures are known, by crystallographic data or in silico procedures.

## 5. Conclusions

IL-6 cytokine early detection is an important tool for the diagnosis and prevention of several diseases characterized by severe inflammatory states, from cancer to COVID-19. The use of Aptamers or Fabs as biosensing elements is often constrained by the specific skills of the researchers and the general requirements of rapid implementation and cost effectiveness. The present theoretical investigation suggests that a new generation aptamer, SOMAmer SL1025, and a Camelid Fab, 61H7, are potentially very good candidates for the production of an IL-6 biosensor. Testing our prediction in a fully-fledged experimental setup seems worth exploring and could enlarge the available set of feasible IL-6 diagnostic tools.

## Figures and Tables

**Table 2:** Table of topological complementarity index, ToCI, using four different contact positions. Lowercase s (#) denotes the ToCI value, calculated putting the input/output contacts on IL-6 helices (#). Data refer to the *rr* spectra reported in Figure 6.

Receptor	s(A-D)	s(A-D*)	s(A-C)	s(B)
IL-6R & gp130	**2.7**	3.4	3.3	3.0
gp130	**4.1**	4.4	4.9	5.1
IL-6R	3.1	**3.0**	5.2	3.5
OKZ-Fab	7.4	**1.9**	5.3	5.7
SL1025-Apt	**1.6**	3.0	2.2	2.4
61H7-Fab	**1.2**	6.7	4.4	4.2
68F2-Fab	**3.4**	4.2	4.4	4.1

**Table 3:** Resistance data for the set of analyzed complexes. The resistances of the complex and the receptor, calculated in the ideal conditions of maximum response, *r_comp_* (*D_1_*), and the limit of detection (LOD), *r_rec_*(*D_LOD_*), normalized to the value of r_rec_(*D_LOD_*) of IL-6R, are reported as well as the corresponding receptor increment Δ, and ratio κ.

Receptor	*r_comp_*(*D_1_*)	*r_rec_*(*D_LOD_*)	κ	Δ/N
IL-6R	16.4	1	16.4	15.4
OKZ-Fab	88.0	0.32	275	86.1
SL1025-Apt	227	15.9	14.3	211
61H7-Fab 68F2-Fab	200 35.9	0.38 0.33	530 110	200 35.9

## Appendix A

The analyzed receptors bind IL-6 in a quite different way. A list of the 3D structures of the cytokine and the different complexes is given in Figure A1.

In a realistic device, electrodes connect the biosensing element (receptors) to the electronic transducer. Therefore, the electrical response due to the binding process has been simulated putting the electrodes *on the receptor*, specifically on the first and last element.

In doing so, we calculated the relative resistance of the complex vs. the receptor, *rr = r_complex_/r_receptor_* (Figure A2). Recall that the receptor is evaluated in its active state. In Figure A2 we report the spectra of relative resistance for the set of analyzed receptors, and (green dashed line) the ratio of the resistance of the IL-6∙ IL-6R complex to the resistance of IL-6R in its native state (PDB ID: 1n26 [47]). As argued in Section 3.2, by increasing *D*, this ratio converges to the *rr* of the IL-6∙ IL-6R complex (black line).

As discussed in Section 3.2, and reported in Figure A2, we focused our attention on the *sensitive D* region, i.e., from the maximum of *rr* to 90% of the difference between the maximum of *rr* and its asymptotic value, here marked with a dot.
